# RACIAL-ETHNIC DIFFERENCES IN MULTIMORBIDITY PROGRESSION ACCORDING TO BODY-WEIGHT STATUS AMONG OLDER U.S. ADULTS

**DOI:** 10.1093/geroni/igz038.1163

**Published:** 2019-11-08

**Authors:** Anda Botoseneanu, Sheila Markwardt, Heather Allore, Corey Nagel, Jason T Newsom, David A Dorr, Ana R Quiñones

**Affiliations:** 1 University of Michigan, Dearborn, Michigan, United States; 2 Oregon Health & Science University, Portland, Oregon, United States; 3 Yale University, New Haven, Connecticut, United States; 4 University of Arkansas for Medical Sciences, Little Rock, Arkansas, United States; 5 Portland State University, Portland, Oregon, United States; 6 Family Medicine, Oregon Health & Science University, Portland, Oregon, United States

## Abstract

Obesity and multimorbidity are more prevalent among underrepresented U.S. racial/ethnic minority groups. Evaluating whether racial/ethnic disparities in multimorbidity accumulation vary according to body-mass index (BMI) may guide interventions aimed at reducing multimorbidity burden in vulnerable racial/ethnic groups. We used 1998-2014 data from the Health & Retirement Study (N=8,635 participants, age 51-55 years old at baseline) and negative binomial models stratified by BMI category to evaluate differences in rates of accumulation of seven chronic conditions (arthritis, cancer, diabetes, heart disease, hypertension, lung disease, and stroke), focusing on differences between racial/ethnic groups [White (reference; 64.7%), Black (21.5%), Hispanic (13.8%)]. Overweight and obesity were more prevalent in Black (80.9%) and Hispanic (78.6%) than White (69.9%) participants at baseline; in all BMI categories, Black participants had higher rates of multimorbidity compared with White participants (normal BMI:β=0.304, p<0.001; overweight:β=0.243,p<0.001; and obese:β=0.135,p=0.013); initial burden of disease was similar between Whites and Hispanics in the normal and overweight categories, but significantly lower among Hispanics (vs. Whites) in the obese category (β= -0.180,p=0.017). We found no significant differences in rates of disease accumulation between the racial/ethnic groups in any of the BMI categories. There are substantial differences in initial disease burden between Black and White middle-aged/older adults, but not in the rate of accumulation of disease between the race/ethnic groups in the 3 main BMI categories. These findings suggest an opportunity to reduce racial disparities in multimorbidity by intervening early in the lifecourse to reduce the burden of chronic disease among vulnerable racial minorities prior to entering middle-age.

